# High-Intensity Physical Training in the Treatment of Chronic Diseases and Disorders

**DOI:** 10.1155/2014/927304

**Published:** 2014-05-05

**Authors:** Lars L. Andersen, David G. Behm, Nicola A. Maffiuletti, Brad J. Schoenfeld

**Affiliations:** ^1^National Research Centre for the Working Environment, Lersø Parkallé 105, DK-2100 Copenhagen O, Denmark; ^2^Memorial University of Newfoundland, St. John's, NL, Canada A1B 3X9; ^3^Schulthess Clinic, 8008 Zurich, Switzerland; ^4^CUNY Lehman College, New York, NY 10065, USA

Chronic diseases, such as diabetes, stroke, heart disease, cancer, and chronic respiratory diseases, are the leading cause of mortality worldwide, accounting for two-thirds of all deaths. Musculoskeletal disorders, such as osteoarthritis, rheumatoid arthritis, osteoporosis, neck pain, and low back pain, are associated with disability, loss of productivity at work, and sick leave. Neurological disorders can affect both physical and mental function and lead to major disability and suffering. In recent years, high-intensity physical training, such as high-intensity cardiovascular training or strength training, has become increasingly popular in rehabilitation of many of these chronic diseases and disorders. However, the efficacy and safety of such high-intensity physical training in the prevention and treatment of chronic diseases and disorders still need to be explored.

In this special issue we invited researchers to contribute original research articles as well as review articles investigating the role of high-intensity physical training in the treatment of chronic diseases and disorders. A wide array of topics is discussed in this special issue, including (1) recovery after ACL reconstruction, (2) chronic kidney disease, (3) frail elderly, (4) hypertension, (5) inflammatory bowel disease, (6) degenerative spinocerebellar disease, (7) chronic pain in the neck and shoulders, (8) obesity, (9) cognitive impairment, (10) substance use disorder, and (11) cardiometabolic risk factors.

Our goal was to touch on different aspects of high-intensity physical training in the treatment of chronic diseases and disorders. We are delighted to see the outcome of the special issue and hope that it will inspire and stimulate further research in this area.

